# Dimethyl 2-(1-benzyl-2-oxoindolin-3-yl­idene)-1,3-dithiole-4,5-dicarboxyl­ate

**DOI:** 10.1107/S1600536811002613

**Published:** 2011-01-22

**Authors:** Ayoob Bazgir

**Affiliations:** a Department of Chemistry, Islamic Azad University, Dooroud Branch, Dooroud 688173551, Iran

## Abstract

In the title compound, C_22_H_17_NO_5_S_2_, the dithiole and oxindole rings are almost coplanar [dihedral angle = 2.71 (8)°] and the phenyl ring makes a dihedral angle of 73.65 (5)° with the oxindole ring. Inter­molecular π–π contacts between adjacent oxindole and dithiole rings [centroid–centroid distance = 3.7273 (11) Å] stabilize the crystal packing.

## Related literature

For the superconducting and optical and electronic switching properties of derivatives of sulfur heterocycles such as thio­phene and 1,3-dithiole, see: Marcos *et al.* (1997 ▶). For the use of 1,3-dithiol-2-ylidenes as building blocks for electronic materials due to their highly electron-donating properties, see: Segura & Martin (2001 ▶). 
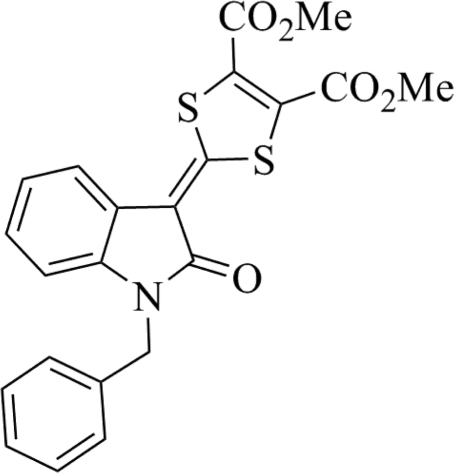


## Experimental

### 

#### Crystal data

C_22_H_17_NO_5_S_2_
*M* *_r_* = 439.51
Monoclinic, 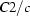

*a* = 28.923 (2) Å
*b* = 9.3615 (5) Å
*c* = 15.0531 (12) Å
β = 101.319 (6)°
*V* = 3996.6 (5) Å^3^
*Z* = 8
Mo *K*α radiation
μ = 0.30 mm^−1^
*T* = 298 K
0.49 × 0.4 × 0.35 mm

#### Data collection

Bruker SMART CCD area-detector diffractometer
Absorption correction: multi-scan (*SADABS*; Bruker, 1998 ▶) *T*_min_ = 0.869, *T*_max_ = 0.900
14881 measured reflections
5379 independent reflections
4206 reflections with *I* > 2σ(*I*)
*R*_int_ = 0.043

#### Refinement

*R*[*F* ^2^ > 2σ(*F* ^2^)] = 0.047
*wR*(*F* ^2^) = 0.113
*S* = 1.07
5379 reflections
271 parameters
H-atom parameters constrained
Δρ_max_ = 0.28 e Å^−3^
Δρ_min_ = −0.20 e Å^−3^

### 

Data collection: *SMART* (Bruker, 1998 ▶); cell refinement: *SAINT* (Bruker, 1998 ▶); data reduction: *SAINT*; program(s) used to solve structure: *SHELXS97* (Sheldrick, 2008 ▶); program(s) used to refine structure: *SHELXL97* (Sheldrick, 2008 ▶); molecular graphics: *ORTEP-3 for Windows* (Farrugia, 1997 ▶); software used to prepare material for publication: *WinGX* (Farrugia, 1999 ▶).

## Supplementary Material

Crystal structure: contains datablocks global, I. DOI: 10.1107/S1600536811002613/bt5465sup1.cif

Structure factors: contains datablocks I. DOI: 10.1107/S1600536811002613/bt5465Isup2.hkl

Additional supplementary materials:  crystallographic information; 3D view; checkCIF report

## Figures and Tables

**Fig. 1.**
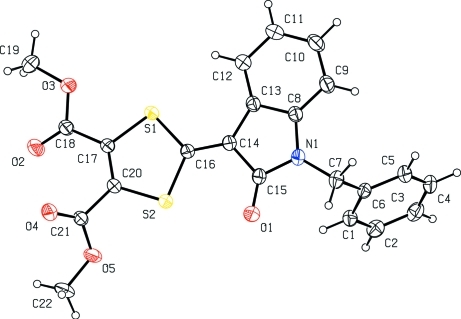
The molecular structure of the title molecule, with the atom-numbering scheme. Displacement ellipsoids are drawn at the 50% probability level.

## supplementary crystallographic information

### Comment

Derivatives of sulfur heterocycles such as thiophene and 1,3-dithiole have been widely explored   because of their superconducting and optical and electronic switching properties (Marcos *et al.*, 1997). 1,3-Dithiol-2-ylidenes have attracted much attention as building blocks for electronic materials due to their highly electron-donating properties (Segura *et al.*,  2001).  In the title compound, the bond distances and angles are within normal ranges. The dihedral angles between the rings A (C8—C15/N1),   B (C16/C17/C20/S1/S2) and   C (C1—C6) are: A/B = 2.71 (6)°, A/C = 73.65 (5)° and B/C = 75.57 (6)°.  Two molecules (symmetry operator: 1-x, y, 0.5-z) are connected by π–π interactions between adjacent A and B rings with a centroid···centroid distance of 3.727Å).

### Experimental

To a magnetically stirred solution of carbon disulfide (1 mmol) and tributylphosphine (1 mmol) in CH_2_Cl_2_ (5 ml) were added benzyl isatin (1 mmol) and dimethyl acetyenedicarboxylate (1 mmol) at room temperature. The mixture was stirred for 2.5 h. After completion of the reaction (TLC), the reaction mixture was filtered off and the residue was washed with ether (10 ml) to afford the pure product as a yellow powder (yield 90%, 0.395 g), mp 451–453 K.

### Refinement

All H atoms were positioned geometrically, with C—H=0.97 Å, 0.96Å and 0.93Å for CH_2_, methyl and aromatic hydrogen atoms, respectively, and constrained to ride on their parent atoms with *U*_iso_(H)=1.2*U*_eq_(C).

### Crystal data

**Table:**

C_22_H_17_NO_5_S_2_	*F*(000) = 1824
*M**_r_* = 439.51	*D*_x_ = 1.461 Mg m^−^^3^
Monoclinic, *C*2/*c*	Mo *K*α radiation, λ = 0.71073 Å
Hall symbol: -C 2yc	Cell parameters from 14881 reflections
*a* = 28.923 (2) Å	θ = 2.3–29.3°
*b* = 9.3615 (5) Å	µ = 0.30 mm^−^^1^
*c* = 15.0531 (12) Å	*T* = 298 K
β = 101.319 (6)°	Block, yellow
*V* = 3996.6 (5) Å^3^	0.49 × 0.4 × 0.35 mm
*Z* = 8	

### Data collection

**Table:**

Bruker SMART CCD area-detector diffractometer	5379 independent reflections
graphite	4206 reflections with *I* > 2σ(*I*)
Detector resolution: 0.15 mm pixels mm^-1^	*R*_int_ = 0.043
φ and ω scans	θ_max_ = 29.3°, θ_min_ = 2.3°
Absorption correction: multi-scan (*SADABS*; Bruker, 1998)	*h* = −39→39
*T*_min_ = 0.869, *T*_max_ = 0.900	*k* = −12→12
14881 measured reflections	*l* = −15→20

### Refinement

**Table:**

Refinement on *F*^2^	Primary atom site location: structure-invariant direct methods
Least-squares matrix: full	Secondary atom site location: difference Fourier map
*R*[*F*^2^ > 2σ(*F*^2^)] = 0.047	Hydrogen site location: inferred from neighbouring sites
*wR*(*F*^2^) = 0.113	H-atom parameters constrained
*S* = 1.07	*w* = 1/[σ^2^(*F*_o_^2^) + (0.0447*P*)^2^ + 2.5812*P*] where *P* = (*F*_o_^2^ + 2*F*_c_^2^)/3
5379 reflections	(Δ/σ)_max_ = 0.007
271 parameters	Δρ_max_ = 0.28 e Å^−^^3^
0 restraints	Δρ_min_ = −0.20 e Å^−^^3^

### Special details

**Table:**

Geometry. All e.s.d.'s (except the e.s.d. in the dihedral angle between two l.s. planes) are estimated using the full covariance matrix. The cell e.s.d.'s are taken into account individually in the estimation of e.s.d.'s in distances, angles and torsion angles; correlations between e.s.d.'s in cell parameters are only used when they are defined by crystal symmetry. An approximate (isotropic) treatment of cell e.s.d.'s is used for estimating e.s.d.'s involving l.s. planes.
Refinement. Refinement of *F*^2^ against ALL reflections. The weighted *R*-factor *wR* and goodness of fit *S* are based on *F*^2^, conventional *R*-factors *R* are based on *F*, with *F* set to zero for negative *F*^2^. The threshold expression of *F*^2^ > σ(*F*^2^) is used only for calculating *R*-factors(gt) *etc*. and is not relevant to the choice of reflections for refinement. *R*-factors based on *F*^2^ are statistically about twice as large as those based on *F*, and *R*- factors based on ALL data will be even larger.

### Fractional atomic coordinates and isotropic or equivalent isotropic displacement parameters (Å^2^)

**Table:**

	*x*	*y*	*z*	*U*_iso_*/*U*_eq_	
C1	0.69401 (6)	0.1560 (2)	0.15830 (13)	0.0440 (4)	
H1	0.6678	0.097	0.1428	0.053*	
C2	0.72780 (7)	0.1575 (2)	0.10498 (15)	0.0517 (5)	
H2	0.7243	0.0992	0.0541	0.062*	
C3	0.76654 (7)	0.2450 (3)	0.12692 (16)	0.0553 (5)	
H3	0.7892	0.2459	0.0909	0.066*	
C4	0.77168 (7)	0.3309 (2)	0.20198 (18)	0.0574 (6)	
H4	0.7978	0.3906	0.2165	0.069*	
C5	0.73822 (7)	0.3290 (2)	0.25619 (16)	0.0505 (5)	
H5	0.7422	0.3866	0.3075	0.061*	
C6	0.69882 (6)	0.24178 (19)	0.23467 (12)	0.0387 (4)	
C7	0.66189 (6)	0.2389 (2)	0.29233 (13)	0.0463 (4)	
H7A	0.6588	0.142	0.3132	0.056*	
H7B	0.6722	0.2985	0.3452	0.056*	
C8	0.60243 (6)	0.4311 (2)	0.22905 (13)	0.0405 (4)	
C9	0.62848 (7)	0.5531 (2)	0.25334 (15)	0.0512 (5)	
H9	0.6597	0.548	0.2835	0.061*	
C10	0.60660 (8)	0.6838 (2)	0.23143 (17)	0.0575 (5)	
H10	0.6235	0.7675	0.2473	0.069*	
C11	0.56030 (8)	0.6924 (2)	0.18661 (17)	0.0579 (6)	
H11	0.5464	0.7813	0.173	0.069*	
C12	0.53431 (7)	0.5688 (2)	0.16160 (15)	0.0495 (5)	
H12	0.5031	0.5747	0.1311	0.059*	
C13	0.55522 (6)	0.43703 (19)	0.18238 (13)	0.0400 (4)	
C14	0.53962 (6)	0.28985 (19)	0.16840 (13)	0.0394 (4)	
C15	0.57937 (6)	0.1980 (2)	0.20878 (13)	0.0413 (4)	
C16	0.49754 (6)	0.23430 (18)	0.12636 (12)	0.0371 (4)	
C17	0.41162 (6)	0.19850 (18)	0.04219 (13)	0.0383 (4)	
C18	0.36382 (7)	0.2325 (2)	−0.01156 (13)	0.0432 (4)	
C19	0.30976 (8)	0.4193 (3)	−0.0566 (2)	0.0695 (7)	
H19A	0.285	0.3699	−0.0351	0.083*	
H19B	0.3077	0.3992	−0.1198	0.083*	
H19C	0.3066	0.5202	−0.0482	0.083*	
C20	0.42817 (6)	0.06699 (18)	0.06540 (13)	0.0375 (4)	
C21	0.40009 (6)	−0.06847 (18)	0.05586 (13)	0.0396 (4)	
C22	0.40263 (9)	−0.3108 (2)	0.01813 (17)	0.0588 (6)	
H22A	0.3728	−0.3055	−0.0232	0.071*	
H22B	0.3978	−0.3414	0.0765	0.071*	
H22C	0.4227	−0.3779	−0.0043	0.071*	
S1	0.450578 (16)	0.33875 (5)	0.07467 (4)	0.04225 (12)	
S2	0.486715 (15)	0.05076 (5)	0.12000 (4)	0.04242 (12)	
O1	0.58087 (5)	0.06729 (15)	0.21051 (11)	0.0533 (4)	
O2	0.33748 (6)	0.14837 (16)	−0.05296 (13)	0.0725 (5)	
O3	0.35496 (5)	0.37174 (14)	−0.00634 (11)	0.0525 (4)	
O4	0.36255 (5)	−0.08135 (16)	0.07518 (12)	0.0573 (4)	
O5	0.42460 (5)	−0.17201 (14)	0.02619 (11)	0.0524 (4)	
N1	0.61588 (5)	0.28852 (17)	0.24442 (12)	0.0434 (4)	

### Atomic displacement parameters (Å^2^)

**Table:**

	*U*^11^	*U*^22^	*U*^33^	*U*^12^	*U*^13^	*U*^23^
C1	0.0381 (9)	0.0446 (9)	0.0469 (10)	−0.0022 (7)	0.0021 (8)	0.0000 (8)
C2	0.0504 (11)	0.0573 (12)	0.0468 (11)	0.0068 (9)	0.0084 (9)	−0.0006 (9)
C3	0.0413 (10)	0.0644 (13)	0.0628 (13)	0.0087 (9)	0.0166 (9)	0.0166 (11)
C4	0.0320 (9)	0.0552 (12)	0.0831 (16)	−0.0061 (8)	0.0068 (9)	0.0046 (11)
C5	0.0383 (9)	0.0501 (11)	0.0589 (12)	−0.0047 (8)	−0.0011 (8)	−0.0086 (9)
C6	0.0309 (7)	0.0408 (9)	0.0418 (9)	0.0015 (7)	0.0007 (7)	0.0032 (7)
C7	0.0383 (9)	0.0576 (11)	0.0415 (10)	−0.0002 (8)	0.0040 (8)	−0.0015 (9)
C8	0.0372 (8)	0.0424 (9)	0.0431 (10)	−0.0050 (7)	0.0105 (7)	−0.0057 (8)
C9	0.0435 (10)	0.0526 (11)	0.0573 (12)	−0.0109 (9)	0.0092 (9)	−0.0128 (10)
C10	0.0586 (12)	0.0452 (11)	0.0689 (15)	−0.0154 (9)	0.0131 (11)	−0.0132 (10)
C11	0.0613 (13)	0.0379 (10)	0.0732 (15)	−0.0043 (9)	0.0100 (11)	−0.0069 (10)
C12	0.0459 (10)	0.0418 (10)	0.0596 (13)	−0.0018 (8)	0.0071 (9)	−0.0027 (9)
C13	0.0365 (8)	0.0414 (9)	0.0433 (10)	−0.0053 (7)	0.0104 (7)	−0.0036 (7)
C14	0.0341 (8)	0.0379 (9)	0.0470 (10)	−0.0029 (7)	0.0095 (7)	−0.0004 (7)
C15	0.0333 (8)	0.0435 (9)	0.0483 (10)	−0.0029 (7)	0.0111 (7)	−0.0009 (8)
C16	0.0342 (8)	0.0344 (8)	0.0433 (9)	−0.0015 (6)	0.0091 (7)	−0.0014 (7)
C17	0.0370 (8)	0.0330 (8)	0.0427 (10)	−0.0013 (6)	0.0026 (7)	−0.0023 (7)
C18	0.0430 (9)	0.0373 (9)	0.0459 (10)	−0.0031 (7)	0.0003 (8)	0.0002 (8)
C19	0.0529 (12)	0.0568 (13)	0.0889 (19)	0.0120 (10)	−0.0108 (12)	0.0065 (13)
C20	0.0364 (8)	0.0334 (8)	0.0423 (9)	−0.0039 (6)	0.0064 (7)	−0.0023 (7)
C21	0.0397 (9)	0.0334 (8)	0.0429 (9)	−0.0042 (7)	0.0011 (7)	0.0008 (7)
C22	0.0763 (15)	0.0299 (9)	0.0697 (15)	−0.0083 (9)	0.0127 (12)	−0.0028 (9)
S1	0.0392 (2)	0.0304 (2)	0.0537 (3)	−0.00321 (17)	0.00062 (19)	0.00031 (19)
S2	0.0350 (2)	0.0320 (2)	0.0588 (3)	−0.00095 (16)	0.00545 (19)	0.00117 (19)
O1	0.0452 (7)	0.0397 (7)	0.0737 (10)	−0.0010 (6)	0.0083 (7)	0.0042 (7)
O2	0.0622 (9)	0.0455 (8)	0.0922 (13)	−0.0049 (7)	−0.0281 (9)	−0.0075 (8)
O3	0.0453 (7)	0.0376 (7)	0.0671 (10)	0.0032 (5)	−0.0078 (7)	−0.0009 (6)
O4	0.0440 (7)	0.0472 (8)	0.0828 (11)	−0.0089 (6)	0.0174 (7)	−0.0041 (7)
O5	0.0579 (8)	0.0310 (6)	0.0709 (10)	−0.0048 (6)	0.0192 (7)	−0.0061 (6)
N1	0.0318 (7)	0.0463 (8)	0.0517 (9)	−0.0019 (6)	0.0068 (6)	−0.0033 (7)

### Geometric parameters (Å, °)

**Table:**

C1—C2	1.381 (3)	C12—H12	0.93
C1—C6	1.387 (3)	C13—C14	1.452 (2)
C1—H1	0.93	C14—C16	1.360 (2)
C2—C3	1.375 (3)	C14—C15	1.469 (3)
C2—H2	0.93	C15—O1	1.224 (2)
C3—C4	1.371 (3)	C15—N1	1.378 (2)
C3—H3	0.93	C16—S1	1.7297 (18)
C4—C5	1.383 (3)	C16—S2	1.7458 (18)
C4—H4	0.93	C17—C20	1.342 (2)
C5—C6	1.387 (2)	C17—C18	1.493 (2)
C5—H5	0.93	C17—S1	1.7368 (17)
C6—C7	1.503 (3)	C18—O2	1.184 (2)
C7—N1	1.460 (2)	C18—O3	1.334 (2)
C7—H7A	0.97	C19—O3	1.446 (2)
C7—H7B	0.97	C19—H19A	0.96
C8—C9	1.377 (3)	C19—H19B	0.96
C8—N1	1.397 (2)	C19—H19C	0.96
C8—C13	1.409 (2)	C20—C21	1.498 (2)
C9—C10	1.387 (3)	C20—S2	1.7376 (18)
C9—H9	0.93	C21—O4	1.184 (2)
C10—C11	1.378 (3)	C21—O5	1.329 (2)
C10—H10	0.93	C22—O5	1.441 (2)
C11—C12	1.391 (3)	C22—H22A	0.96
C11—H11	0.93	C22—H22B	0.96
C12—C13	1.382 (3)	C22—H22C	0.96
			
C2—C1—C6	120.58 (18)	C8—C13—C14	106.16 (16)
C2—C1—H1	119.7	C16—C14—C13	130.87 (17)
C6—C1—H1	119.7	C16—C14—C15	121.66 (16)
C3—C2—C1	120.2 (2)	C13—C14—C15	107.46 (15)
C3—C2—H2	119.9	O1—C15—N1	125.82 (17)
C1—C2—H2	119.9	O1—C15—C14	128.00 (17)
C4—C3—C2	119.9 (2)	N1—C15—C14	106.17 (15)
C4—C3—H3	120	C14—C16—S1	123.04 (14)
C2—C3—H3	120	C14—C16—S2	122.44 (14)
C3—C4—C5	120.24 (19)	S1—C16—S2	114.52 (10)
C3—C4—H4	119.9	C20—C17—C18	125.59 (16)
C5—C4—H4	119.9	C20—C17—S1	116.20 (13)
C4—C5—C6	120.5 (2)	C18—C17—S1	118.16 (13)
C4—C5—H5	119.7	O2—C18—O3	124.74 (18)
C6—C5—H5	119.7	O2—C18—C17	125.09 (18)
C1—C6—C5	118.57 (18)	O3—C18—C17	110.17 (15)
C1—C6—C7	120.09 (16)	O3—C19—H19A	109.5
C5—C6—C7	121.34 (18)	O3—C19—H19B	109.5
N1—C7—C6	113.04 (16)	H19A—C19—H19B	109.5
N1—C7—H7A	109	O3—C19—H19C	109.5
C6—C7—H7A	109	H19A—C19—H19C	109.5
N1—C7—H7B	109	H19B—C19—H19C	109.5
C6—C7—H7B	109	C17—C20—C21	126.35 (16)
H7A—C7—H7B	107.8	C17—C20—S2	117.91 (13)
C9—C8—N1	128.91 (17)	C21—C20—S2	115.54 (13)
C9—C8—C13	121.75 (18)	O4—C21—O5	125.71 (17)
N1—C8—C13	109.33 (15)	O4—C21—C20	124.68 (17)
C8—C9—C10	117.89 (19)	O5—C21—C20	109.56 (15)
C8—C9—H9	121.1	O5—C22—H22A	109.5
C10—C9—H9	121.1	O5—C22—H22B	109.5
C11—C10—C9	121.45 (19)	H22A—C22—H22B	109.5
C11—C10—H10	119.3	O5—C22—H22C	109.5
C9—C10—H10	119.3	H22A—C22—H22C	109.5
C10—C11—C12	120.4 (2)	H22B—C22—H22C	109.5
C10—C11—H11	119.8	C16—S1—C17	96.21 (8)
C12—C11—H11	119.8	C20—S2—C16	95.02 (8)
C13—C12—C11	119.46 (19)	C18—O3—C19	115.73 (17)
C13—C12—H12	120.3	C21—O5—C22	115.50 (16)
C11—C12—H12	120.3	C15—N1—C8	110.87 (15)
C12—C13—C8	119.08 (17)	C15—N1—C7	123.44 (16)
C12—C13—C14	134.76 (17)	C8—N1—C7	125.68 (16)
			
C6—C1—C2—C3	−0.4 (3)	S1—C17—C18—O2	164.3 (2)
C1—C2—C3—C4	0.1 (3)	C20—C17—C18—O3	166.85 (19)
C2—C3—C4—C5	0.6 (3)	S1—C17—C18—O3	−15.7 (2)
C3—C4—C5—C6	−0.9 (3)	C18—C17—C20—C21	−10.8 (3)
C2—C1—C6—C5	0.1 (3)	S1—C17—C20—C21	171.74 (15)
C2—C1—C6—C7	−179.80 (18)	C18—C17—C20—S2	174.62 (16)
C4—C5—C6—C1	0.5 (3)	S1—C17—C20—S2	−2.9 (2)
C4—C5—C6—C7	−179.58 (19)	C17—C20—C21—O4	−43.9 (3)
C1—C6—C7—N1	−63.5 (2)	S2—C20—C21—O4	130.81 (19)
C5—C6—C7—N1	116.6 (2)	C17—C20—C21—O5	138.6 (2)
N1—C8—C9—C10	−178.9 (2)	S2—C20—C21—O5	−46.6 (2)
C13—C8—C9—C10	0.8 (3)	C14—C16—S1—C17	−178.17 (17)
C8—C9—C10—C11	−0.1 (3)	S2—C16—S1—C17	2.04 (12)
C9—C10—C11—C12	−0.4 (4)	C20—C17—S1—C16	0.44 (17)
C10—C11—C12—C13	0.3 (4)	C18—C17—S1—C16	−177.23 (15)
C11—C12—C13—C8	0.4 (3)	C17—C20—S2—C16	3.65 (17)
C11—C12—C13—C14	179.3 (2)	C21—C20—S2—C16	−171.53 (14)
C9—C8—C13—C12	−0.9 (3)	C14—C16—S2—C20	177.01 (17)
N1—C8—C13—C12	178.79 (18)	S1—C16—S2—C20	−3.19 (12)
C9—C8—C13—C14	179.82 (18)	O2—C18—O3—C19	−0.7 (3)
N1—C8—C13—C14	−0.4 (2)	C17—C18—O3—C19	179.30 (19)
C12—C13—C14—C16	1.8 (4)	O4—C21—O5—C22	−0.4 (3)
C8—C13—C14—C16	−179.2 (2)	C20—C21—O5—C22	177.06 (17)
C12—C13—C14—C15	−178.9 (2)	O1—C15—N1—C8	178.81 (19)
C8—C13—C14—C15	0.2 (2)	C14—C15—N1—C8	−0.5 (2)
C16—C14—C15—O1	0.3 (3)	O1—C15—N1—C7	−1.8 (3)
C13—C14—C15—O1	−179.1 (2)	C14—C15—N1—C7	178.90 (17)
C16—C14—C15—N1	179.60 (17)	C9—C8—N1—C15	−179.7 (2)
C13—C14—C15—N1	0.2 (2)	C13—C8—N1—C15	0.6 (2)
C13—C14—C16—S1	1.5 (3)	C9—C8—N1—C7	0.9 (3)
C15—C14—C16—S1	−177.74 (14)	C13—C8—N1—C7	−178.76 (18)
C13—C14—C16—S2	−178.71 (16)	C6—C7—N1—C15	101.1 (2)
C15—C14—C16—S2	2.0 (3)	C6—C7—N1—C8	−79.6 (2)
C20—C17—C18—O2	−13.2 (4)		
